# Acute Effects of Global Postural Re-Education on Non-Specific Low Back Pain. Does Time-of-Day Play a Role?

**DOI:** 10.3390/ijerph18020713

**Published:** 2021-01-15

**Authors:** David Merinero, Manuel Rodríguez-Aragón, Javier Álvarez-González, Álvaro López-Samanes, Joaquín López-Pascual

**Affiliations:** 1 Exercise Physiology Group, School of Physiotherapy, Faculty of Health Sciences, Universidad Francisco de Vitoria, 28223 Madrid, Spain; davidmerinero@gmail.com (D.M.); j.alvarezglez.prof@ufv.es (J.Á.-G.); alvaro.lopez@ufv.es (Á.L.-S.); 2 Departamento de Economía de la Empresa, Universidad Rey Juan Carlos, Vicálvaro, 28032 Madrid, Spain; joaquin.lopez@urjc.es

**Keywords:** chronobiology, physical therapy, treatment

## Abstract

Low back pain is one the most common forms of musculoskeletal disorders. Thus, several physiotherapeutic strategies (e.g., global postural re-education therapy) have been used for reducing low back pain. The aim of this study was to determinate if acute application of global postural re-education session associated effects are influenced by the time-of-day when this physical therapy is applied. Eight participants in a randomized, counterbalanced order were acutely tested both before and 24 h after a global postural re-education therapy session (10 min session) in three different time-of-day points; morning (i.e., AM; 7:00–9:00 h), midday (i.e., AM; 12:00–14:00 h) and afternoon (i.e., PM; 18:00–20:00 h). In each session, low back pain Visual Analogue Pain Scale [VAS]), flexibility, function capacity (Roland Morris Questionnaire [RMQ], and physical functioning Oswestry Disability Index [ODI]) were recorded. Results showed a pain reduction (VAS Scale) 24 h post Global postural re-education [GPR] session (*p* = 0.001) and increasing of flexibility pre-post GPR session in all the time-of-day points (morning, midday, and afternoon) (*p* = 0.001) while no differences were reported in RMQ (*p* = 0.969) and ODI (*p* = 0.767). Thus, acute GPR session produces the same effects on flexibility, low back pain, function capacity, and physical functioning values independently of time-of-day when it is applied.

## 1. Introduction

Low back pain[LBP] is normally defined as muscle tension or pain located below the costal margin and above the inferior gluteal folds, associated with or without pain in the lower-limbs [1], being one of the most prevalent musculoskeletal disorders reported worldwide [2]. The etiology of this pathology is multifactorial, reporting different risk factors englobed in two different categories; modifiable (e.g., falls, inactivity, smoking) and non-modifiable factors (e.g., age, gender, genetics, prior work exposures) [3]. In addition, this pathology reported high incidence, mentioning that 75–85% of the individuals will experience low back pain during their lifetime with a high prevalence of recurrent episodes [4]. Thus, different physiotherapeutic methods have been proposed in the treatment of low back pain such as electrotherapy, massotherapy, pilates, therapeutic exercise, or global postural re-education [5,6,7].

Global postural re-education (GPR) is a physical therapy method developed in France in the 1980s by therapist Phillipe Souchard [8]. This therapeutic method is based upon the idea that the muscular system is formed by muscle chains and combines global stretching postures with breathing and proprioceptive techniques with the aim of stretching the shortened muscles to restore the excess tension and pain produced in the musculoskeletal system [9,10]. Based on the growing interest of this physical therapy method, recent interest in the scientific community has been founded with different research groups studying the efficacy of this method in different pathologies such as chronic neck pain [11], urinary incontinency [12,13], ankylosing spondylitis [14,15,16], spinal disorders [17], temporomandibular disorders [18], and more recently, their effects in sports performance [19]. However, some issues need to be addressed in the research field of global postural re-education such as differences between acute/chronic application of this physical therapy, differences between sexes (male versus females), or even differences when this treatment is applied at different times-of-day points, and the influence of circadian rhythms.

Circadian rhythms are the biological variations that occur in each life form in the 24 h periods that are regulated by exogenous (e.g., light and temperature), sleep-wake cycles (e.g., mental fatigue), and endogenous mechanisms (e.g., changes in body internal system) [20]. These circadian rhythms are regulated by a central “clock” called suprachiasmatic nucleus, located in the hypothalamus and which is responsible for regulating a multitude of physiological functions that occur in the body [21]. In addition to this central regulator, other secondary clocks regulate other structures highlighting skeletal muscle [22], tendons [23], cartilage and intervertebral discs [24]. Thus, changes in flexibility values (e.g., flexion and extension low back movements) have been reported previously during the day [25].

However, to our knowledge, this is the first study that has analyzed the influence of applying a GPR program at different time-of-day points on pain, flexibility, and quality-of-life parameters on non-specific low back pain. The aim of this study was to determinate if acute effects of global postural re-education session associated effects are influenced by the time-of-day when this physical therapy is applied.

## 2. Materials and Methods

### 2.1. Participants

Eight participants (six female and two male) (age, 27.4 ± 8.4 years; body mass, 65.2 ± 7.8 kg; height, 1.65 ± 0.1 m; body mass index, 23.9 ± 2.7; and physical activity > 3 days per week of regular physical activity) volunteered to participate in this investigation. All the participants were informed of the tests they were to perform and signed the consent form. The experimental procedure of this study was conducted in accordance with the Declaration of Helsinki and the approval by the Ethics Committee of the University Francisco de Vitoria, number 20/2019.

### 2.2. Study Design

The recruitment process was realized by university workers via different social networks. Thirty-four participants were selected and only thirteen were recruited to participate in the study based on the exclusion/inclusion criteria. Once the study started, five participants were unable to complete the three experimental sessions due to different reasons such as illness (*n* = 1), unexpected problems with their work schedules (*n* = 3), and unexpected issues without formal explications (*n* = 1). Thus, finally, eight participants took part in this study. The inclusion criteria were: (a) age between 18 and 40 years old; (b) chronic non-specific lower back pain characterized by mechanical pain over a period of more than three months; (c) score of 3.0 to 10.0 on a 0–10 visual analog scale for pain (this range of scores was chosen to allow analysis of changes that might occur in this parameter). Exclusion criteria were: (a) acute and subacute lower back pain; (b) specific causes of low back pain (e.g., herniated disc, lumbar stenosis, spinal deformity, fracture, spondylolisthesis); (c) central or peripheral neurological signs; (d) systemic diseases (e.g., tumor and rheumatological diseases) and psychiatric mental disorders; (e) patients who had undergone other surgical interventions within the 6 months prior to the initial assessment. One researcher (A.L.S.) allocated all the participants’ in a randomized crossover design using the Research Randomizer (www.randomizer.org) The randomization was realized by an investigator that did not take part in GPR session measurements and data analysis. In addition, participants completed three identical testing sessions in three different days with one week between to allow a full recovery.

### 2.3. Experimental Procedure

**Figure 1:**
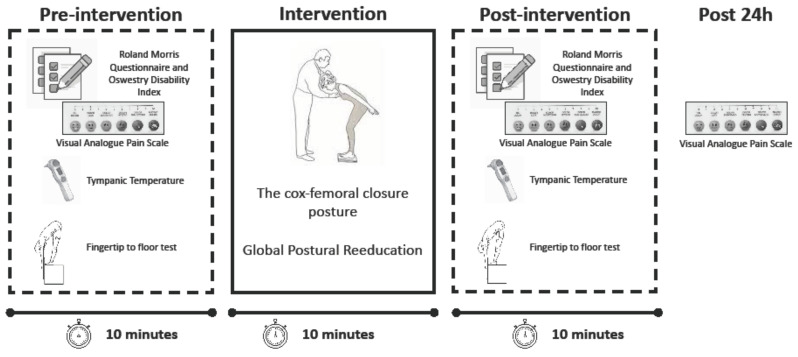
Experimental design.

In a randomized counter-balance order, study participants underwent the same global postural re-education session in the morning (AM), midday (MD), or in the afternoon (PM): (a) AM protocol started with a morning session between 7:00–9:00 h, (b) MD protocol with a session between 12:00–14:00, and (c) PM protocol between 18:00–20:00. The chosen time-of-day point was selected according to the Ethics and Methods Consensus for Biological Rhythms Research on animals and human beings, establishing that during chronobiology studies, at least three time points need to be carried out for a correct design [26]. Three testing sessions were realized in the study (9:00 h, 14:00 h, and 20:00 h). The day before beginning the first experimental testing, participants’ height was measured to the nearest 0.5 cm using a wall mounted stadiometer (Seca 202, Seca Ltd., Hamburg, Germany) and body composition was assessed by bioimpedance (Tanita BC-601, Tanita Corp., Tokyo, Japan). For all the GPR sessions, participants arrived to the testing facilities 10 min before testing to fill out an Roland Morris Questionnaire [RMQ] and Oswestry Disability Index [ODI] questionnaire and report Visual Analogue Pain Scale [VAS] pain values. Before and after each GPR session, air temperature and tympanic temperature were measured with a portable weather station (Meteorological Station, Küken, Spain) and a tympanic thermometer (Braun Thermoscan IRT6520, Braun, Germany) reporting similar environmental (air temperature 25.4 ± 0.5 °C) and tympanic temperature conditions (36.1 ± 0.6 °C) between trials. For GPR treatment, the cox-femoral closure posture was used. It is an active exercise by the patient where the posture evolves from a position that seeks a static alignment, with the feet placed one centimeter away from one heel to the other and an opening angle between the first toes of 30 °C. The knees remain semi-flexed and in a neutral hip position. The arms are placed extended along the body with a slight abduction and external rotation. The positioning criteria of the spinal column include an alignment between the occipital, the eighth dorsal vertebra, and the second sacral vertebra, respecting physiological lordosis and kyphosis. Once this alignment is obtained, a hip flexion is performed slowly and progressively (cox-femoral closure) and the parameters described above are maintained [8]. The therapist guides the patient’s progression by simple verbal commands or by giving tactile references in the areas of failure, or misalignment. The treatment session lasted ten minutes per subject, depending of the endurance of each patient, two breaks of twenty seconds each are permitted during treatment [7]. Immediately, after the intervention, RMQ/ODI questionnaire VAS scale were reported, additionally 24 h post VAS reports were requested. (Figure 1).

### 2.4. Fingertip to Floor Test

While standing barefoot, heels on the floor, feet at shoulder width, and knees straight in platform of 20 cm with shoes removed and feet close together, the participants were asked to bend forward as far as possible, while maintaining their knees and arms fully extended. Distance from the tip of the middle finger to the floor was measured with a semirigid metal tape measure and recorded to the distance in cm [27].

### 2.5. Visual Analogue Scale (VAS)

VAS in a pain rating scale, in which scores are based on self-reported measures of symptoms that are recorded with a single handwritten mark placed at one point along the length of a 10 cm line that represents a continuum between the two ends of the scale—“no pain” on the left end (0 cm) of the scale and the “worst pain” on the right end of the scale (10 cm) [28].

### 2.6. Roland Morris Questionnaire [RMQ] and Oswestry Disability Index (ODI)

Disability was assessed by following self-administered evaluation scales: the Roland and Morris Disability Questionnaire and the Oswestry Disability Index (ODI). The RMQ is validated in Spanish [29] and comprises 24 items in which greater levels of disability are reflected by higher numbers on a 24-point scale [30]. The RMQ has been shown to yield reliable measurements, which are valid for inferring the level of disability, and to be sensitive to change over time for groups of patients with low back pain LBP [31]. The ODI, which was used in Spanish, is structured in 10 sections corresponding to different daily activities, each scored on a six-point scale (0–5).

### 2.7. Statistical Analysis

Data are presented as mean ± standard deviation (SD). Data were inspected visually and statistically for normality (Shapiro–Wilk’s test, *p*-value > 0.05), and all variables were normally distributed. VAS was analyzed by 2-way repeated measures ANOVA with condition (morning (7:00–9:00) vs. midday (12:00–14:00) vs. afternoon (18:00–20:00) and time (pre-test, post-test, and 24 h post-test). For RMQ and ODI 2 (time: pre-test and post-test)) × 3 (condition: morning (7:00–9:00) vs. midday (12:00–14:00) vs. afternoon (18:00–20:00), analysis of variance (ANOVA) was performed to examine the effects of different conditions. The effect size (Cohen’s d)—the difference between pretest and posttest means divided by their common standard deviation were calculated and interpreted as small (d = 0.2), medium (d = 0.5) or large d = 0.8) to present the magnitude of the effect. [32]. All data analyses were performed using SPSS software (version 20.0, IBM, Armonk, NY, USA).

## 3. Results

### 3.1. Fingertip to Floor Test

**Figure 2:**
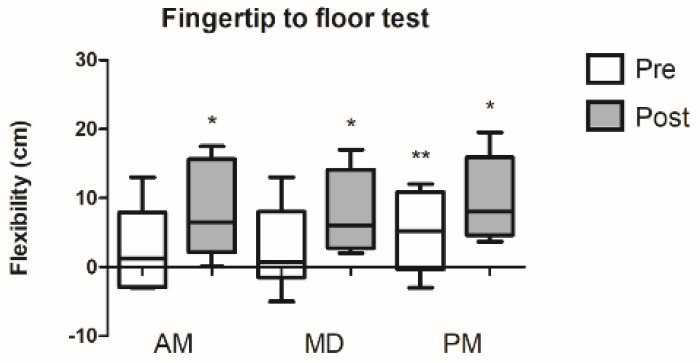
Flexibility values in morning, midday, and evening conditions. * Significant differences compared to the AM values at *p* ≤ 0.05, ** Significant differences compared PM pre vs. AM/MD M post values at *p* ≤ 0.05.

For flexibility values measured by fingertip to floor test, the condition × time interaction (*p* = 0.787) was not significant. However, a significant main effect of time (*p* = 0.003) and condition (*p* = 0.044) was observed (Figure 2).

### 3.2. Low Back Pain

**Figure 3:**
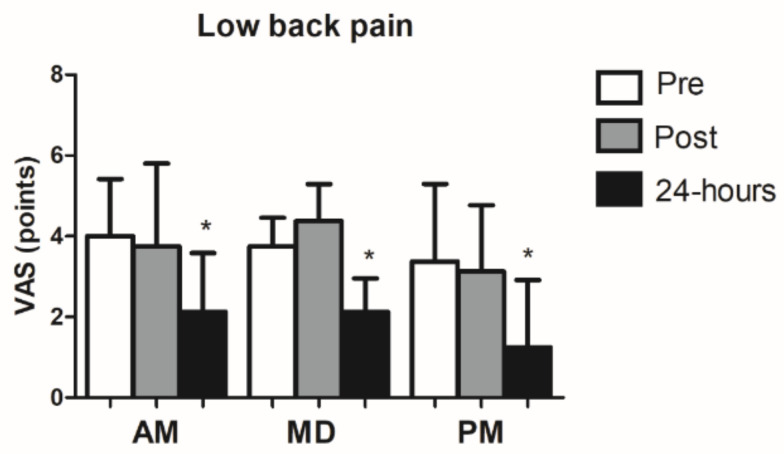
Low back pain values in morning, midday, and evening conditions. * Significant differences compared to the AM values at *p* ≤ 0.05.

For low back pain measured by VAS scale, the condition × time interaction (*p* = 0.339), and main effect of condition (*p* = 0.109) were not significant. However, a significant main effect of time (*p* = 0.001) was observed, indicating a difference between pre and 24 h intervention VAS scale. (Figure 3).

### 3.3. Roland Morris Questionnaire (RMQ) and Oswestry Disability Index (ODI)

For the RMQ, the condition × time interaction (*p* = 0.693) main effect of condition (*p* = 0.346) and the main effect of time were not significant (*p* = 0.969), (Table 1). According to the ODI test, the condition × time interaction (*p* = 0.521) main effect of condition (*p* = 0.151) and the main effect of time were not significant (*p* = 0.777).

## 4. Discussion

This study analyzed the effects of circadian rhythms on flexibility, low back pain, physical functioning, and function capacity after the GPR therapy application. According to this objective, we assessed the effects of GPR of three separate time-of-day points (i.e., (7:00–9:00) vs. midday (12:00–14:00) vs. afternoon (18:00–20:00)) and their effects on different health parameters (e.g., pain). The main finding of our study was acute treatment of GPR produces the same effects independently of time-of-day when it is applied. Therefore, to our knowledge, no previous studies have analyzed the effects of time-of-day when GPR therapy is applied on flexibility, pain, function capacity, and physical functioning.

According to this study, we observed a decrease in pain perception measured by VAS scale in individuals with nonspecific low-back pain after one session of GPR (24 h post treatment), independently of the time-of-day when the therapy was applied (morning vs. midday vs. evening) It is worth to mention that all of the participants obtained a punctuation scored above 6 points. In addition, our data is in agreement with previous studies developed by Cunha et al. [11] that reported lower VAS values after two sessions per week of GPR during a six-week period of treatment and Cavalcanti [33] who reported statistical differences after 10 sessions during a 6-week period. However, according to our data, it seems that the application of only one GPR session provoked the same effects during different time-of-day points. Therefore, a need for long-term effects studies applying GPR therapy particularly for patients with chronic low back pain should be developed.

Regarding flexibility, GPR reported an increment of all conditions after pre-post evaluations (morning, midday, or afternoon) without reaching statistical significance between time-of-day points. This increase, could be attributed to different factors provoked by GPR application such as an improvement in viscoelastic properties and increases of sarcomeres in series [11], influence of stretching on muscle spindles/joint receptors [34], or body temperature increments [35] that could contribute to an increase of nerve conductivity, increasing actin-myosin cross bridging processes [36], phosphagen metabolism, or muscle buffering capacity [37]. In addition, our finding corroborates previous studies that showed flexibility improvements after RPG treatment in chronic low-back patients [38] or even in basketball athletes [39].

Non-specific low back pain has been related to emotional factors, thus, several questionnaires for measuring these items in non-specific low back pain have been developed. Thus, according to our data, no statistical differences were reported in RMQ and ODI pre-post GPR intervention. Our data are contradictory comparing previous results obtained by different research groups [38,40]. However, the discrepancies between studies could be attributed to the length of the different studies; Bonetti et al. (2010) studied the effects of applying GPR therapy during six months and Castagnoli et al. (2015) analyzed the chronic effects of applying GPR during a year´s follow-up study, while our study is focused on observing the acute effects of GPR after one session. Furthermore, the different GPR positions used in the studies could explain the differences in our results, comparing the studies previously mentioned.

The current study presents some potential limitations that should be mentioned. A first limitation is the reduced sample size used in this study. For this reason, we should be careful when drawing any definitive conclusions as small samples. A second limitation is that we only studied the effect of GPR therapy in unexperienced patients with this physical therapy method and in isolation, showing their effects on flexibility, pain, and disability in patients with non-specific low back pain. Thus, future research studies should determine the effects of applying GPR in isolation and in combination with other physical therapy methods such as therapeutic exercise. A third limitation of this study was the acute application of GPR therapy (three sessions). A fourth limitation of this study was that the authors could not analyze the relationship between chronotype and time-of-day when the GPR treatment is applied due to the limited sample size. A fifth limitation is the lack of control group. Thus, the outcomes of this investigation should be only translated to unexperienced GPR patients that received acute application of GPR on three time-of-day different points.

## 5. Conclusions

The current results showed that acute GPR session produces the same effects on flexibility, low back pain, function capacity, and physical functioning values independently of time-of-day when it is applied.

## Figures and Tables

**Table 1:** Outcomes for morning, midday, and afternoon conditions.

Variables	Fingertip to Floor Test	RMQ	ODI
Morning (7:00–9:00)			
Pre	2.68 ± 6.01	3.50 ± 2.83	11.75 ± 5.39
Post	8.18 ± 7.12	3.88 ± 3.73	11.00 ± 7.17
%Change	75.40 ± 58.70	5.25 ± 11.73	7.13 ± 18.09
ES	0.83	0.11	0.12
Midday (12:00–14:00)			
Pre	2.69 ± 5.96	4.50 ± 3.93	13.50 ± 6.99
Post	7.94 ± 5.84	4.38 ± 4.37	12.25 ± 7.67
%Change	39.30 ± 31.30	1.85 ± 3.71	11.03 ± 23.60
ES	0.88	0.03	0.17
Afternoon (18:00–20:00)			
Pre	5.19 ± 5.78	3.75 ±4.13	10.00 ± 7.78
Post	9.90 ± 5.99	3.63 ± 4.03	10.25 ± 8.45
%Change	55.71 ± 57.05	1.48 ± 8.53	1.38 ± 3.09
ES	0.80	0.03	0.03

Abbreviations: RMQ = Roland Morris Questionnaire; ODI = Oswestry Disability Index; ES = effect size.

## Data Availability

The data presented in this study are available on request from the corresponding author.
